# The Effect of Long-Term Inorganic Iodine on Intrathyroidal Iodothyronine Content and Gene Expression in Mice with Graves' Hyperthyroidism

**DOI:** 10.1089/thy.2022.0496

**Published:** 2023-03-16

**Authors:** Toyoyoshi Uchida, Mika Shimamura, Hikari Taka, Naoko Kaga, Yoshiki Miura, Yuya Nishida, Yuji Nagayama, Hirotaka Watada

**Affiliations:** ^1^ Department of Metabolism & Endocrinology, Juntendo University Graduate School of Medicine, Tokyo, Japan.; ^2^ Department of Molecular Medicine, Atomic Bomb Disease Institute, Nagasaki University, Nagasaki, Japan.; ^3^ Laboratory of Proteomics and Biomolecular Science, Biomedical Research Core Facilities, Juntendo University Graduate School of Medicine, Tokyo, Japan.

**Keywords:** Graves' disease, inorganic iodine, iodothyronine, RNA sequencing

## Abstract

**Background:**

The main molecular mechanism underlying acute suppression of iodine organification in normal thyroids after an excessive iodine load, that is, the Wolff–Chaikoff effect, is assumed to be suppression of iodine oxidation and iodothyronine synthesis. However, the mechanism underlying chronic antithyroid action of inorganic iodine in Graves' disease is not fully understood. Using a mouse model of Graves' hyperthyroidism, we examined changes in iodothyronine content and gene expression profiles in the thyroid glands after inorganic iodine loading.

**Materials and Methods:**

Graves' hyperthyroidism was induced and maintained in BALB/c mice by repeated immunizations of recombinant adenovirus expressing the human thyrotropin (TSH) receptor A-subunit. Hyperthyroid mice were left untreated (GD-C; *n* = 8) or treated with inorganic iodine for 12 weeks (GD-NaI; *n* = 8). We used unimmunized BALB/c mice as a control group (*n* = 10). In each mouse, serum thyroxine (T4) levels were measured with enzyme-linked immunosorbent assay (ELISA) at 4-week intervals. The intrathyroidal iodothyronine content and gene expression levels were, respectively, evaluated by mass spectrometry and RNA sequencing (RNA-seq) at the end of the experimental period.

**Results:**

Serum T4 levels in the GD-C group remained higher than in the control group, whereas those in the GD-NaI group declined to normal levels during the experimental period. Intrathyroidal triiodothyronine (T3), reverse T3 (rT3), and T4 contents in the GD-C group were higher than the control group, and rT3 and T4 were further increased in the GD-NaI group. The observed alterations in iodothyronine levels in the thyroid and sera may be explained by altered expression levels of genes for iodothyronine biosynthetic molecules, their transporter, and deiodinases.

**Conclusion:**

In this mouse model of hyperthyroidism, higher intrathyroidal accumulation of T4 and reduced gene expression data of iodothyronine transporters in the GD-NaI group suggest that chronic antithyroid action of iodine in Graves' disease involves suppression of hormone secretion.

## Introduction

Iodine is an essential component of thyroid hormones. Thyroid epithelial cells actively incorporate iodine through the sodium/iodide symporter (NIS) in the basolateral membrane and concentrate it. Iodine is then transferred to the follicular lumen through other iodine transporters, such as pendrin (PDS), which is located in the apical membrane. Iodine is oxidized and incorporated into tyrosine residues of thyroglobulin (Tg) by thyroid peroxidase (TPO) and in the presence of H_2_O_2_ generated by dual oxidase 2 (DUOX2), a member of the NADPH oxidase family, to form monoiodotyrosine (T1) and diiodotyrosine (T2). Subsequently, T1 and T2 are coupled to produce triiodothyronine (T3) and thyroxine (T4).

Tg with these iodothyronines is reabsorbed through endocytosis into thyroid epithelial cells through the microvilli in the apical membrane. Tg is then subjected to proteolysis by cathepsins in lysosome to release free T3 and T4. Some T4 is converted into T3 or reverse T3 (rT3) by iodothyronine deiodinases (DIO). These hormones are then secreted into the circulation by hormone transporters such as monocarboxylate transporter 8 (MCT8).^1^ Furthermore, iodine in remnant Tg iodotyrosines is reused for hormone production through iodotyrosine dehalogenase 1. In the normal thyroid, these pathways are positively regulated by thyrotropin receptor (TSHR) signaling. In Graves' disease, these signals are enhanced by thyroid stimulating antibodies (TSAbs).^1–3^

Excessive inorganic iodine has therapeutic effects in patients with Graves' hyperthyroidism^4–6^ and may be safely used in those with pregnancy and lactation.^7,8^ The Wolff–Chaikoff effect is assumed to suppress iodine oxidation and then iodothyronine synthesis.^9–13^ Yet, clinical research on the effects of long-term inorganic iodine administration in patients with Graves' disease has demonstrated suppressed iodine oxidation with increased intrathyroidal iodothyronine levels.^14^ The suppression of thyroid hormone secretion is a major contributor to the antithyroid effects of iodine.^15,16^ However, the molecular mechanism explaining these findings is not known.

In this study, we examined the effects of inorganic iodine on iodothyronine levels in a mouse model of Graves' hyperthyroidism and analyzed iodine-induced alterations in the expression of genes coding molecules related to thyroid function with RNA sequencing (RNA-seq) analysis.

## Materials and Methods

### Animals and experimental procedures

Six-week-old BALB/c (H-2d) male and female mice were purchased from Charles River Breeding Laboratories (Tokyo, Japan). Mice were kept under specific pathogen-free conditions throughout the experiments, maintained in a 12-hour light/dark cycle, and fed a standard rodent diet (CE2; Japan CLEA, Tokyo, Japan) and water *ad libitum*. Animal care and all experimental procedures were performed in accordance with the Guideline for Animal Experimentation of Nagasaki University with approval of the institutional animal care and use committee (permission No. 1803191437). All surgeries were performed under isoflurane anesthesia, and every effort was made to minimize suffering.

**FIG. 1.**
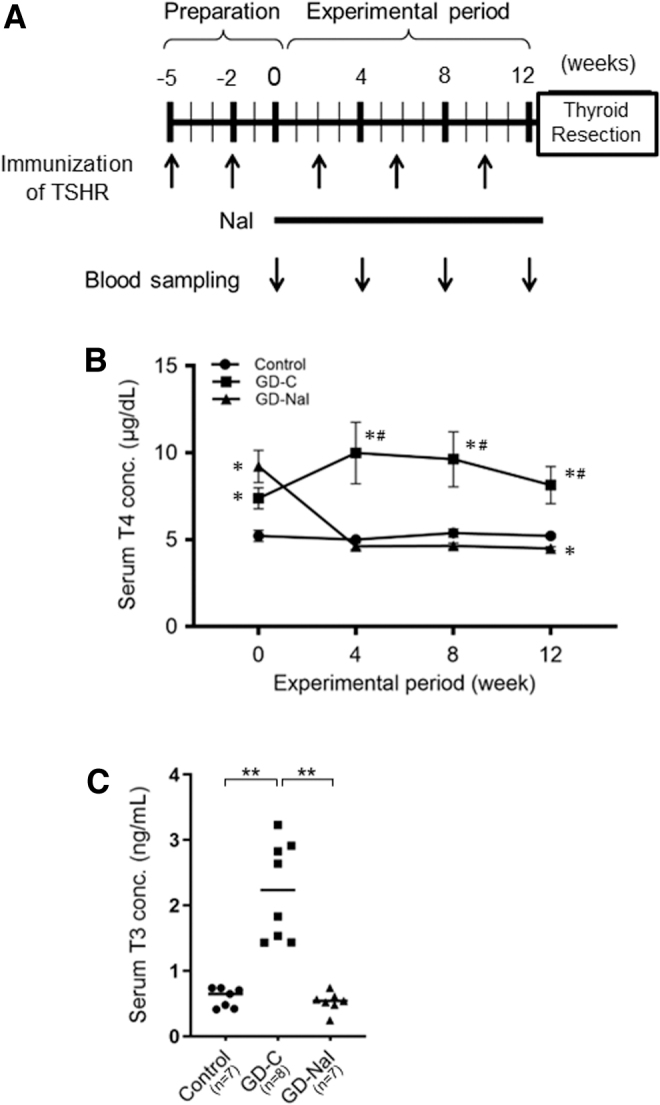
The effect of inorganic iodine administration in a mouse model of Graves' disease with hyperthyroidism. **(A)** The experimental protocol. **(B)** Sequential serum T4 measurements in control mice (the control group, *n* = 10), the nontreated group (the GD-C group, *n* = 8), and the iodine-treated group, which received water containing 0.05% sodium iodine (the GD-NaI group, *n* = 8). Mice with hyperthyroidism at week 0 (the GD-C and GD-NaI groups) were immunized twice before the 12-week experimental period. **(C)** Serum T3 measurements in respective groups at 12 weeks. The mean values are shown by horizontal lines. *p < 0.05, **p < 0.01 relative to control group. ^#^*p* < 0.05, **p < 0.01 relative to the GD-NaI group. T3, triiodothyronine; T4, thyroxine.

Figure 1A demonstrates the experimental protocol. Thirty-four mice were immunized with recombinant adenovirus expressing the human TSHR A subunit (Ad-hTSHR A subunit)^17^ twice at a 3-week interval (weeks 5 and 2) and then thrice at a 4-week interval (weeks 2, 6, and 10). Blood was obtained every 4 weeks (weeks 0, 4, 8, and 12) for T4 measurements. Two weeks after the second immunization, 16 mice (8 male and 8 female) were found to be hyperthyroid. They were divided into two groups. The GD-NaI group was treated with 0.05% sodium iodine in drinking water for 12 weeks (*n* = 8). The GD-C group was untreated (*n* = 8).

At week 12, the mice were sacrificed and the thyroids were removed. One lobe was kept in RNAlater (Qiagen, Valencia, CA) for RNA-seq. The other lobe was kept in −80°C for liquid chromatography–mass spectrometry (LC/MS). Control mice (*n* = 10) were untreated for the entire experimental period.

### High-performance LC/MS

T1, T2, T3, rT3, and T4 were extracted from mouse thyroid tissue according to the methods described by Ackermans et al.^18^ In brief, frozen thyroid tissue was homogenized in methanol containing 100 fmol of T3-^13^C_6_, rT3-^13^C_6_, and 1 pmol of T4-^13^C_6_ as internal standards. After centrifugation, the supernatant was dried with nitrogen stream and stored at −80°C until LC/MS analysis. Intrathyroidal T1, T2, T3, rT3, and T4 contents were analyzed with the Nexera LC system connected to LCMS-8060 tandem mass spectrometer (Shimadzu, Kyoto, Japan).

Samples were dissolved in 50% MeOH and analyzed with the LC/MS system. They were separated using L-column3, which is a 3 μm, 15 cm × 2.1 mm column (Chemicals Evaluation and Research Institute, Saitama, Japan). The temperature was set at 40°C for T3, rT3, and T4 analyses and at 25°C for T1 and T2 analyses. High performance Liquid Chromatography solvents consisted of (A) 0.1% formic acid/H_2_O and (B) 0.1% formic acid/MeOH. The gradient program was 50% B for 2 minutes, increased by 2.5%/min to 95% B, and stayed at 95% B for 10 minutes at a flow rate of 150 μL/min for T3, rT3, and T4 analyses.

For T1 and T2 analyses, the gradient began at 20% B for 3 minutes, increased by 2%/min to 95% B, and stayed at 95% B for 10 minutes at a flow rate 100 μL/min. MS analysis was carried out in multiple reaction monitoring positive ionization mode. Iodothyronine contents were corrected by protein content for comparisons among groups.

### T4 and T3 measurements

Serum total T4 and T3 concentrations in mice were measured using commercially available enzyme-linked immunosorbent assay (ELISA) kits (LifeSpan BioSciences, Inc., Seattle, WA and Thermo Fisher Scientific, Waltham, MA, respectively). The normal range of T4 was defined as the mean ± 3 standard deviation of control mice (*n* = 10).

### TSAb measurements

TSAb activity in mouse serum was measured using a commercially available kit using porcine thyroid cells (TSAb kit; Yamasa EIA, Chiba, Japan) and a bioluminescent cAMP assay (cAMP-Glo™ Assay; Promega, Madison, WI). TSAb (%) was calculated by dividing cAMP concentrations of mice in the GD-C or the GD-NaI groups by cAMP concentrations of mice in the control group. TSAb >150% was judged as positive.

### RNA sequencing

Total RNA in the thyroid (*n* = 4 mice per group) was extracted using the RNeasy Plus Mini Kit (Qiagen). RNA quality was tested using the Agilent 2100 Bioanalyzer (Agilent Technologies, Palo Alto, CA). The SMART-Seq v4 Ultra Low Input RNA Kit for Sequencing (Clontech, Mountain View, CA) was used for complementary DNA (cDNA) preamplification. Sequencing libraries were prepared using the Nextera XT DNA Library Prep Kit (Illumina, San Diego, CA). All samples had a peak size of ∼150 base pairs and were sequenced with the NovaSeq 6000 analyzer (Illumina).

Sequence reads were mapped against the *Mus musculus* genome assembly (Genome Reference Consortium GRCm38, Release M25). DRAGEN (Dynamic Read Analysis for GENomics) Bio-IT Platform version 3.6.3 was used for hierarchical clustering and heatmaps. Gene ontology (GO) and pathway enrichment analysis were performed using DAVID version 6.8^19^

### Reverse transcription polymerase chain reaction and quantitative real-time polymerase chain reaction

The first strand cDNA was synthesized using 0.5 μg of total RNA with SuperScript™ III First-Strand Synthesis SuperMix (Thermo Fisher Scientific). For quantitative real-time polymerase chain reaction (PCR), cDNA was analyzed by the QuantStudio 7 Flex Fast Real-Time PCR System with SYBR Green PCR Master Mix (Thermo Fisher Scientific) and specific primers (Supplementary Table S1). Thermal cycle for amplification was set as follows: 20 seconds at 95°C and 40 cycles of 95°C for 1 second and 60°C for 20 seconds. Using this protocol, neither nonspecific primer–dimmer amplification nor PCR products were observed in no-template control samples.

The relative messenger RNA (mRNA) expression levels of target genes were analyzed using the 2′(–delta delta threshold cycles) method. As an internal invariant standard, we used TATA box binding protein (Tbp) mRNA.

### Statistical analysis

Differences in sequential serum T3, T4 levels, TSAb levels, iodothyronine contents, and relative mRNA expression levels were examined using one-way analysis of variance with the *post hoc* Turkey's test. SPSS version 18.0 for Windows (SPSS, Inc., Chicago, IL) was used for these analyses. Statistical analyses of RNA-seq data were performed using the edgeR package (Bioconductor),^20^ which is optimized for analysis of RNA-seq expression profiles with biological replication between two groups. A *p-*value of <0.05 was considered statistically significant.

## Results

### Amelioration of hyperthyroidism by inorganic iodine in mice

After two immunizations with Ad-hTSHR A subunit, serum T4 levels were significantly higher in the GD-C and GD-NaI groups than in the control group (Fig. 1B). An additional 3 immunizations at a 4-week interval maintained a hyperthyroid state in the GD-C group, but iodine loading decreased T4 concentrations to the basal levels in 4 weeks and maintained to 12 weeks in the GD-NaI group. Serum T3 concentrations at 12 weeks were also significantly higher in the GD-C than in the control group and GD-NaI group (Fig. 1C). Recurrence of hyperthyroidism was not observed in any mice in the GD-NaI group. These data suggest that long-term iodine loading has a suppressive effect on Graves' hyperthyroidism in mice.

### Effect of inorganic iodine on intrathyroidal iodotyrosine/iodothyronine levels in mouse thyroids

**FIG. 2.**
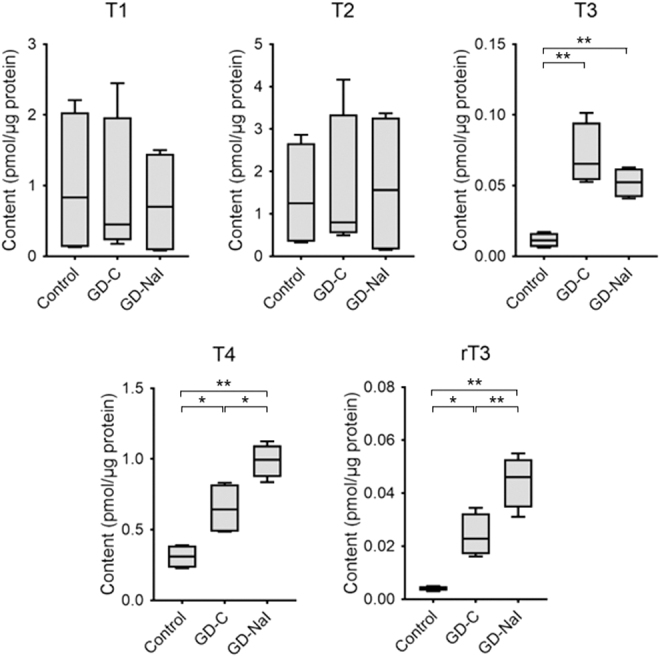
Change in intrathyroidal iodothyronine content. Thyroids were resected after the 12-week experimental period and LC/MS was performed (*n* = 4). Data are presented as boxes (95th percentiles and means) and whiskers (range). *p < 0.05. **p < 0.01. LC/MS, liquid chromatography–mass spectrometry.

Intrathyroidal iodothyronine content was then measured. Intrathyroidal T1 and T2 content was unchanged in all three groups. Compared with the control group, the T3 content increased in the GD-C and GD-NaI groups, and T4 and rT3 content increased in the GD-C group, as well as to a greater extent in the GD-NaI group (Fig. 2).

### Suppression of thyroid hormone biosynthesis by inorganic iodine in Graves' disease

We conducted transcriptome analysis with RNA-seq of thyroid tissues from mice in the control group and mice in the GD-C and the GD-NaI groups that had the highest TSAb levels at 12 weeks (*n* = 4 in each group) (Supplementary Fig. S1). Hierarchical clustering and heatmap analysis demonstrated dynamic changes in gene expression among the three groups (Supplementary Fig. S2). In particular, similar patterns of unbiased clustering in the control and the GD-NaI groups suggested that iodine normalized altered gene expression in the GD-C group. We then focused on upregulated genes in the GD-C group relative to the control group and downregulated genes in the GD-NaI group relative to the GD-C group, to identify genes induced in hyperthyroidism and suppressed by long-term iodine loading.

**FIG. 3.**
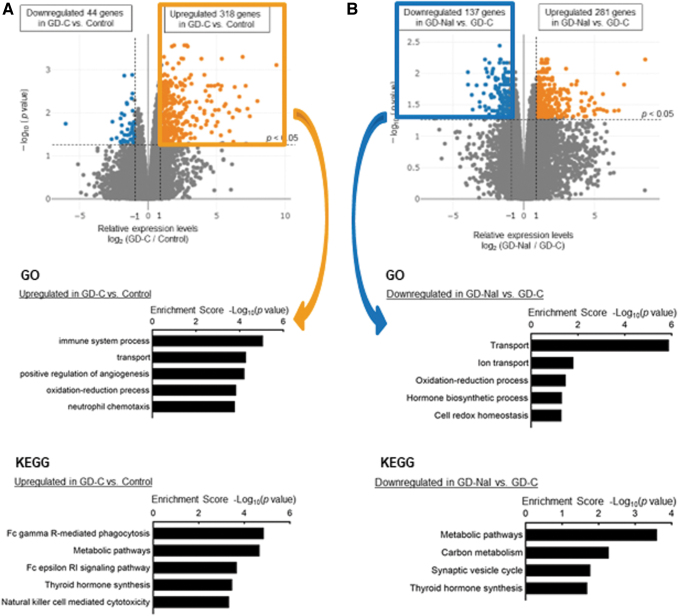
Transcriptome analysis of the thyroid. Thyroids were resected after the 12-week experimental period and RNA-seq was performed (*n* = 4). **(A)** Volcano plot comparing RNA-seq data from the control and GD-C groups (upper panel). The two dashed vertical lines represent the thresholds of log_2_ (fold change; greater than or equal to 1 or less than or equal to −1). GO enrichment analysis was performed for genes that were significantly upregulated in the thyroid of the GD-C group relative to the control group (middle panel). KEGG pathway enrichment analysis was performed for genes that were significantly upregulated in the thyroid of the GD-C group relative to the control group (lower panel). **(B)** Volcano plot of the GD-C and GD-NaI groups (upper panel). GO enrichment analysis was performed for genes that were significantly downregulated in the thyroid of the GD-NaI group relative to the GD-C group (middle panel). KEGG pathway enrichment analysis was performed for genes that were significantly downregulated in the thyroid of the GD-NaI group relative to the GD-C group (lower panel). GO, gene ontology; KEGG, Kyoto Encyclopedia of Genes and Genomes; RNA-seq, RNA sequencing.

Volcano plots revealed that 318 genes were upregulated and 44 genes were downregulated in the GD-C group relative to the control group (Fig. 3A, upper panel); 137 genes were upregulated and 281 genes were downregulated in the GD-NaI group relative to the GD-C group (Fig. 3B, upper panel). Functional annotation with the biological processes of these differentially expressed genes was performed with GO enrichment analysis. The upregulated genes in the GD-C group relative to the control group and the downregulated genes in the GD-NaI group relative to the GD-C group were commonly enriched in transport/ion transport and oxidation–reduction process/cell redox homeostasis (Fig. 3A, middle and 3B, middle).

The Kyoto Encyclopedia of Genes and Genomes (KEGG) analysis identified thyroid hormone synthesis as a significant pathway in the GD-C group relative to the control group (Fig. 3A, lower panel) and in the GD-NaI group relative to the GD-C group (Fig. 3B, lower panel). Thus, genes related to transport, redox balance, and thyroid hormone synthesis were upregulated in Graves' hyperthyroidism, and these changes were negated by long-term iodine loading.

### Suppression of genes involved in thyroid hormone synthesis and transport by inorganic iodine

Table 1 lists genes that were annotated with the aforementioned terms. Genes that appeared twice (*Slco4a1*, *Tpo*, *Dio1*, *Duoxa2*, and *Slc26a4*) and thyroid-specific genes that appeared once (*Tshr*, *Slc5a5*, and *Duox2*) are shown in bold. These findings suggest that the genes related to Graves' hyperthyroidism are *Tshr*, *Tpo*, *Dio1*, and *Slco4a1* (a thyroid hormone transporter). The genes related to the antithyroid effects of long-term iodine loading are *Slc5a5*, *Slc26a4*, *Duox2*, and *Duoxa2*, in addition to *Tpo*, *Dio1*, and *Slco4a1*, which are upregulated in Graves' hyperthyroidism. These findings suggest that long-term iodine loading decreases T4 synthesis by reducing iodine uptake and oxidation capacity.

**Table 1.** Genes Listed in Gene Ontology and Kyoto Encyclopedia of Genes and Genomes Terms That Were Upregulated in the GD-C Group and Downregulated in the GD-NaI Group Relative to the GD-C Group

GO term	Genes involved
Upregulated in the GD-C group relative to the control group	
Transport	*Lgals3*, *Fcgr3*, *Fcer1g*, *Syk*, *Itgb2*, *Nckap1l*, ***Slco4a1***, *Trem1*, *Vav1*
Positive angiogenesis	*Lgals3*, *Ddah1*, *Cma1*, *Prkcb*, *Adm2*, *Sphk1*, *Itgb2*, *C3ar1*, *Cybb*, *Hmox1*, *Aqp1*, *Runx1*
Oxidation–reduction process	*Moxd1*, *Fa2h*, ***Tpo***, *Ero1lb*, ***Dio1***, *Cyp1a1*, *Ndufa1*, *Glrx*, *Sod3*
Downregulated in the GD-NaI group relative to the GD-C group	
Transport	*Lcn8*, *Ero1lb*, *Aqp6*, *Glrx*, *Fmn2*, *Vldlr*, *Snx31*, ***Duoxa2***, *Slc16a9*, ***Slc26a4***, *Slc38a3*, *Slc18a1*, *Gpihbp1*, *Atp6v1c2* *Ndufa1*, *Kcnj16*, *Nme4*, *Tmc7*, *Fa2h*, *Slc5a8*, ***Slco4a1***, *Slco4c1*, *Rbp1*, *Tmem9*, *Folr1*, *Slc25a33*, *Atp6v0d2*, *Hcn2*
Ion transport	*Slc5a8*, ***Slco4a1***, *Slco4c1*, *Kcnj16*, *Tmc7*, *Slc38a3*, *Atp6v0d2*, *Hcn2*, *Atp6v1c2*
Oxidation–reduction process	*Moxd1*, *Fa2h*, ***Tpo***, *Ero1lb*, ***Dio1***, *Cyp1a1*, *Ndufa1*, *Glrx*, *Sod3*
Hormone biosynthetic process	***Tpo***, ***Dio1***
Cell redox homeostasis	*Ero1lb*, *Glrx*
*KEGG term*	*Genes involved*
Upregulated in the GD-C group relative to the control group	
Thyroid hormone synthesis	*Gpx2*, *Prlcb*, ***Tpo***, ***Tshr***
Downregulated in the GD-NaI group relative to the GD-C group	
Thyroid hormone synthesis	***Duox2***, ***Duoxa2***, ***Scl5a5***, ***Slc26a4***, ***Tpo***

Genes that appeared twice and thyroid-specific genes that appeared once are shown in bold.

GO, gene ontology; KEGG, Kyoto Encyclopedia of Genes and Genomes.

However, these changes are apparently contradictory to higher intrathyroidal T4 content. An increase in intrathyroidal T4 contents is assumed to be due to decreased release of iodothyronines, reduced hormone secreting capacity, or both. Based on the RNA-seq results, genes related to liberation of iodothyronines from Tg such as cathepsins were not listed in Table 1. Instead, expression of *Slco4a1* was significantly upregulated in the GD-C group and downregulated in the GD-NaI group. Lower *Slco4a1*gene expression may be related to a reduction in hormone secreting capacity and subsequently intrathyroidal T4 accumulation.

### RNA-seq analysis examining the changes in deiodinases in Graves' hyperthyroidism and in response to inorganic iodine

**FIG. 4.**
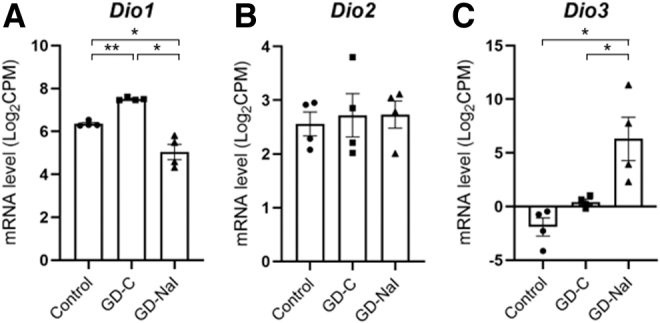
Changes in the expression of intrathyroidal deiodinase genes in mice with hyperthyroid Graves' disease and in response to inorganic iodine administration. **(A)** *Dio1*, **(B)** *Dio2*, and **(C)** *Dio3*. Data are shown as bar graphs with dots indicating data from individual mice (*n* = 4) based on RNA-seq results. Data are presented as means ± SE. *p < 0.05. **p < 0.01. CPM, count per million; SE, standard error.

To address the mechanism for the changes in T3 and rT3 contents (Fig. 2), we evaluated changes in the expression levels of other deiodinases (Fig. 4). Increased T4 synthesis predominantly led to conversion into T3 rather than rT3 with upregulation of *Dio1* gene expression in the GD-C group. T4 that accumulated as a result of long-term iodine loading was predominantly converted into rT3 rather than T3 with upregulation of *Dio3* expression and downregulated *Dio1* gene expression in the GD-NaI group.

### Gene expression evaluated by quantitative real-time PCR in Graves' hyperthyroidism and in response to inorganic iodine

**FIG. 5.**
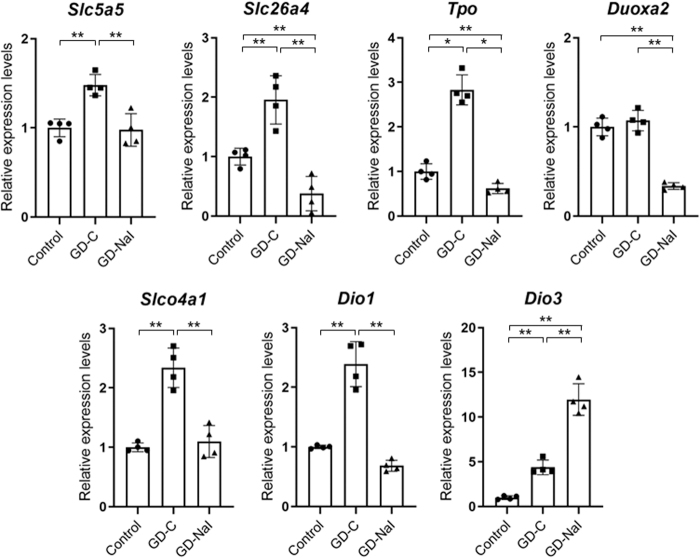
Gene expression levels by RNA-seq and Dio3. Relative mRNA expression levels of *Slc5a5* (NIS), *Slc26a4* (PDS), *Tpo*, *Douxa2*, *Slco4a1* (OAPT4A1), *Dio1*, and *Dio3* determined with quantitative real-time PCR are shown as bar graphs with dots indicating individual data (*n* = 4). Data are presented as the means ± SE. *p < 0.05 and **p < 0.01. mRNA, messenger RNA; NIS, sodium/iodide symporter; PCR, polymerase chain reaction; PDS, pendrin.

To confirm the changes of gene expression levels determined with RNA-seq analysis, quantitative real-time PCR was performed on the samples from the thyroids among the three groups (Fig. 5). Although the differences detected by quantitative real-time PCR were in general smaller than those by RNA-seq, the gene expression levels of *Slc5a5*, *Slc26a4*, *Tpo*, *Slco4a1*, and *Dio1* were significantly increased in the GD-C group and decreased in the GD-NaI group, and those of *Dio3* increased in the GD-C group and further in the GD-NaI group. In addition, that of *Douxa2* was significantly decreased in the GD-NaI group. These findings are consistent with the results of RNA-seq and intrathyroidal iodothyronine content.

## Discussion

In this study, we showed that long-term iodine loading reduced serum T4 concentrations but increased intrathyroidal T4 contents in a mouse model of Graves' hyperthyroidism. This finding is similar to that observed in patients with Graves' hyperthyroidism.^14^ We investigated the change of thyroid gene expression by long-term iodine loading in a mouse model and found that iodine uptake, oxidation, and transport were enhanced in Graves' hyperthyroidism, and suppressed in response to long-term iodine loading.

We observed that intrathyroidal T4 and T3 content increased in mice with Graves' hyperthyroidism. This is possibly due to increasing hormone biosynthesis through stimulation from TSAb and DIO1-mediated T4 to T3 conversion, respectively. Previous studies reported that in the thyroid of patients with untreated Graves' disease, T4 content is slightly higher or equal to that of euthyroid individuals and T3 content is significantly increased; therefore, the intrathyroidal T4/T3 ratio is significantly lower than in euthyroid individuals.^21,22^

This may be due to enhanced deiodination from T4 to T3 through DIO1 in patients with Graves' hyperthyroidism and our data suggest that the DIO1 is activated in Graves' hyperthyroidism. We believe that our mouse model demonstrates analogous intrathyroidal hormone levels to those in patients with Graves' disease.

Our data demonstrate that long-term iodine loading in Graves' hyperthyroidism also induces iodine autoregulation by decreasing the expression of genes involved in iodine uptake and oxidation. Thus, long-term iodine loading is likely to decrease thyroid hormone synthesis. However, we observed that long-term iodine loading induced higher increased intrathyroidal T4 content. In a clinical study, Nagataki et al concluded that iodine treatment (10 mg/day) for 14 days in patients with untreated Graves' disease suppresses thyroid iodine uptake and oxidation, but increases absolute iodine uptake into the thyroid due to higher blood iodine levels and consequently higher T4 content in the thyroid.^14^

Their conclusions are consistent with those of an *in vivo* study that showed that iodine loading markedly increases T4 content in the thyroid of rats, despite suppression of hormone synthesis through the inhibition of iodine oxidation with propylthiouracil pretreatment.^23^ Furthermore, other studies have shown that during inorganic iodine loading, the attenuation rate of exogenous radioisotopes (^131^I) accumulated in the thyroid decreases (both *in vivo* and in patients with Graves' disease), thereby suggesting that the reason for the increased T4 content is not only the absolute iodine load, but also decreased hormone secretion.^15,16,24–26^

Regarding the molecules related to T4 secretion, MCT8 has been considered to be a main transporter of thyroid hormone, although the complete mechanism of T4 secretion is not fully understood.^1,27^ A previous study showed that short-term (1 day) iodine overloading decreases the expression of MCT8 at the mRNA and protein levels, but relatively long-term (6 day) iodine overloading does not.^28^ In our study, long-term iodine loading also did not affect the gene expression of *Slc16a2* (MCT8) in the three groups (*p* = 0.18 [Control vs. GD-C] and 0.87 [GD-C vs. GD-NaI], respectively).

However, our RNA-seq analysis showed that the gene expression of *Slco4a1*, which is also known as organic anion transporting polypeptide (OATP) 4A1, was increased in Graves' hyperthyroidism and decreased with long-term inorganic loading. Previous studies demonstrated that OATP4A1, which can transport thyroid hormones, is expressed in various tissues and its transport capacity is in the order of T3 > T4 = rT3.^29,30^ The biological function of OATP4A1 should be further elucidated in this context.

Finally, long-term iodine loading induced higher *Dio3* gene expression with increased rT3 content, as well as a decreased *Dio1* gene expression with decreased T3 content. In patients with Graves' disease treated with inorganic iodine, serum rT3 concentrations increase,^31,32^ serum T3 concentrations rapidly decrease, and the serum T4/T3 ratio is significantly increased.^33^ These data suggest that DIO1 is involved in iodine autoregulation and DIO3 is sensitive to inorganic iodine.

## Conclusion

This mouse model study demonstrates that long-term iodine loading in Graves' hyperthyroidism suppresses iodine uptake, oxidation, and iodothyronine synthesis, but increases intrathyroidal T4 content (accumulation) through suppression of hormone secretion, which decreases serum thyroid hormone levels. More research is needed to elucidate the transcriptional regulation of iodine-responsive genes in this context.

## Supplementary Material

Supplemental data

Supplemental data

Supplemental data
