# Supplementary material for: The Effect of Long-Term Inorganic Iodine on Intrathyroidal Iodothyronine Content and Gene Expression in Mice with Graves' Hyperthyroidism
Source: Thyroid. 2023 Mar 16;33(3):330–7. doi: 10.1089/thy.2022.0496 (PMC10024588; doi:10.1089/thy.2022.0496)
Supplement: Supplemental data [file Suppl_TableS1.docx]

Supplementary Table 1. Primers used for quantitative real-time PCR

| Genes (accession number) | Forward primer (5’-3’) | Reverse primer (5’-3’) |
| --- | --- | --- |
| Slc5a5 (NM_053248) | GGGATGCACCAATGCCTCTG | GTAGCTGATGAGAGCACCACA |
| Slc26a4 (NM_011867) | GCTCGCATTCGGGACTGTAA | CAGCAAACCTGCTTTGGCAT |
| Tpo (NM_009417) | CAAAGGCTGGAACCCTAATTTCT | AACTTGAATGAGGTGCCTTGTCA |
| Duoxa2 (NM_025777) | CGTTAACATTACACTCCGAGGAACA | CAGAATGCCACCCACAGTGT |
| Dio1 (NM_007860) | CATCTGGGATTTCATTCAAGGC | TGGAGGCAAAGTCATCTACGAGTC |
| Dio3 (NM_172119) | CCGCTCTCTGCTGCTTCAC | CGGATGCACAAGAAATCTAAAAGC |
| Slco4a1 (NM_148933) | GCATTCCAGCGCTAACTGCTAC | TGTCAATCACCCAGCCAAAG |
| Tbp (NM_1004198) | GCTGCAGTCATCATGAGAATAAGAG | CACCATGTTCTGGATCTTGAAGT |
