# Supplementary figures and images for: The Effect of Long-Term Inorganic Iodine on Intrathyroidal Iodothyronine Content and Gene Expression in Mice with Graves' Hyperthyroidism
Source: Thyroid. 2023 Mar 16;33(3):330–7. doi: 10.1089/thy.2022.0496 (PMC10024588; doi:10.1089/thy.2022.0496)

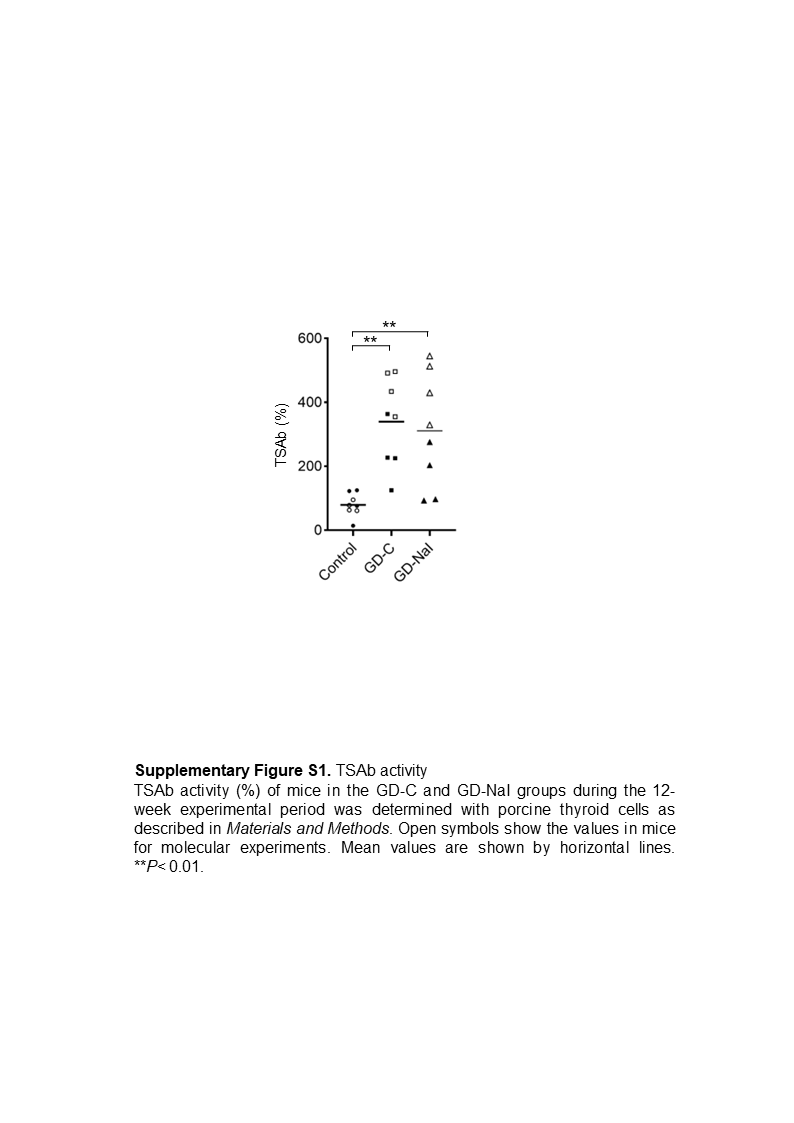

Supplement: Supplemental data [file Suppl_FigS1.tif]

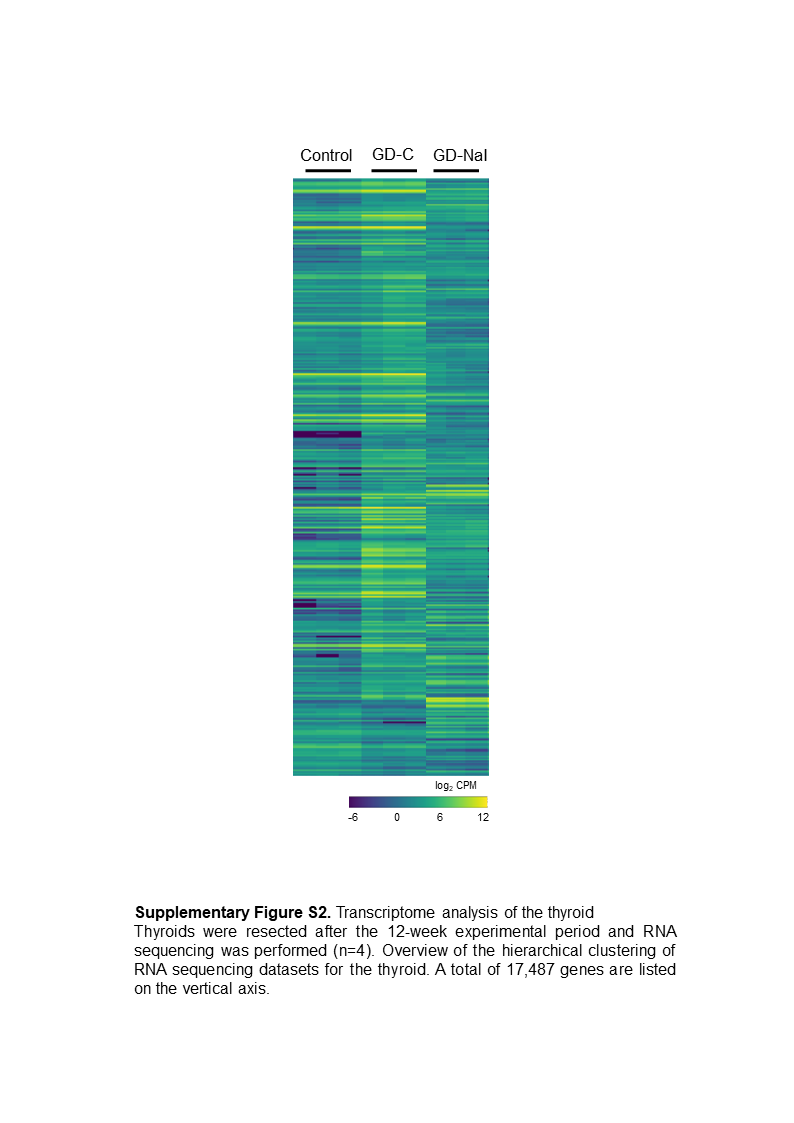

Supplement: Supplemental data [file Suppl_FigS2.tif]
